# Synthesis, Applications and Biological Impact of Nanocellulose

**DOI:** 10.3390/nano12183188

**Published:** 2022-09-14

**Authors:** Rajesh Sunasee, Karina Ckless

**Affiliations:** Department of Chemistry and Biochemistry, State University of New York at Plattsburgh, Plattsburgh, NY 12901, USA

Interest in cellulose-based nanomaterials has continued to increase dramatically in the past few years, especially with advances in the production routes of nanocellulose—such as cellulose nanocrystals (CNC), cellulose nanofibrils (CNF) and bacterial nanocellulose (BNC)—that tailor their performances [1,2,3]. In addition, given the presence of ample hydroxyl groups on the surface of nanocellulose, there have been significant developments in the chemical modifications of nanocellulose toward the design, fabrication and characterization of advanced functional nanocellulose-based materials for various applications [4,5,6,7]. Nanocellulose exhibits unique characteristics (v/s molecular cellulose) due to its nanoscale size, large surface area and other physicochemical properties. Research in the past decade has shown that nanocellulose is a promising bio-based material for several biomedical applications [6,8], given its biocompatibility and relatively low risk to human health. However, to advance the nano-biomedical applications of nanocellulose, it is crucial to develop a solid understanding of the biological impacts of nanocellulose, including toxicity, genotoxicity and potential immune responses elicited by these nanomaterials [9].

The present Special Issue in Nanomaterials aims to highlight recent advances in the synthesis of nanocellulose, surface modifications for the design of functional nanocellulose as well as its applications and potential biological impact. It features two review articles and four original research articles authored and reviewed by experts in the nanocellulose field. The compilation of the reviews and research articles for this book targets a broad readership of chemists, materials scientists, biochemists, nanotechnologists and others with an interest in nanocellulose research.

An extensive review by Lam and Hemraz discusses the preparation and surface functionalization of carboxylated CNC [10]. While sulfated CNC, derived from the sulfuric-acid-mediated hydrolysis of cellulose, has been the predominant form of this class of nanocellulose, carboxylated CNC has emerged as a similar material of interest for various studies and potential applications. The presence of the carboxyl groups on the nanocrystal’s surface enables several chemical modification approaches to be explored for the design of functionalized carboxylated CNC. This review targets the recent progress in methods and feedstock materials for producing carboxylated CNC, their functional properties and discussions on the initial successes in their applications. The authors also discuss some of the inherent advantages that carboxylated CNC might possess in similar applications with sulfated CNC. The second review by Finny et al. focuses on 3D-printable nanocellulose-based functional materials [11]. The authors summarize the potential of nanocellulose as a promising material for designing functional nanostructures and devices via 3D printing. The different properties, preparation methods, printability and strategies to functionalize nanocellulose into 3D-printed constructs are thoroughly discussed. The recent development in 3D-printed nanocellulose-based composites for food, environmental, food packaging, energy, and electrochemical applications is also highlighted.

In an original research study, Shaikh et al. report the preparation of poly(vinyl alcohol)/guar-gum-based phase-separated film, which is then incorporated with date-palm-derived CNC as the reinforcing agent [12]. The films are synthesized via the solution-casting method and characterized by various spectroscopic and microscopic techniques. A drug release study shows that the release of Moxifloxacin can be tailored by altering the CNC content within the phase-separated films. Overall, the developed films are found to be ideal as delivery carriers for Moxifloxacin. The biological impact of nanocellulose is portrayed in three other original research studies [13,14,15]. For instance, Bernier et al. investigated the impact of a library of cationic CNC in the human blood and endothelial cells using cell-based assays [13]. They observe that despite the cationic CNC not changing RBC morphology or causing aggregation, at 24 h exposure, mild hemolysis is detected, mainly with pristine CNC. They further study the effect of various concentrations of CNC on the cell viability of human umbilical vein endothelial cells (HUVECs) in a time-dependent manner. Results indicated that none of the cationic CNCs caused a dose–response decrease in the cell viability of HUVEC at 24 h or 48 h of exposure. Overall, the findings of this study, combined with the authors’ previous study on the immunomodulatory properties of these cationic CNCs [16], support the potential development of engineered cationic CNCs as vaccine nanoadjuvants. 

In another study, Mota et al. describe the use of the fluorescence microscopy technique to detect and visualize BNC and BCNC nanomaterials [14]. Both BNC and BCNC are prepared and characterized, and using adsorption studies, the interaction of a cellulose-binding module fused to a green fluorescent protein (GFP–CBM) with BNC and BCNC is investigated along with the uptake of BCNC by macrophages. An initial in vivo study shows that BNC or BCNC throughout the gastrointestinal tract is only observed in the intestinal lumen, indicating that cellulose particles are not absorbed. Wacker et al. study the effect of surface-coating BNC small-diameter vascular grafts with human albumin, fibronectin or heparin–chitosan upon endothelialization with human saphenous vein endothelial cells (VEC) or endothelial progenitor cells (EPC) in vitro [15]. The results indicate that the fibronectin coating significantly promotes the adhesion and growth of VEC and EPC, while albumin only promotes the adhesion of VECs. The heparin–chitosan coating only significantly improves the adhesion of EPC. Overall, both fibronectin and heparin–chitosan coatings could impact the endothelialization of BNC-SDVGs and, as such, could be promising approaches to help improve the longevity and reduce the thrombogenicity of BNC small-diameter vascular grafts.

Overall, this book compiles two excellent reviews with highlights of challenges and future directions as well as four articles related to research with nanocellulose from the Special Issue entitled “Synthesis, Applications and Biological Impact of Nanocellulose”.

## References

[1] Klemm D., Kramer F., Moritz S., Lindstroem T., Ankerfors M., Gray D., Dorris A. (2011). Nanocelluloses: A New Family of Nature-Based Materials. Angew. Chem. Int. Ed..

[2] Vanderfleet O.M., Cranston E.D. (2021). Production routes to tailor the performance of cellulose nanocrystals. Nat. Rev. Mater..

[3] Reid M.S., Villalobos M., Cranston E.D. (2017). Benchmarking Cellulose Nanocrystals: From the Laboratory to Industrial Production. Langmuir.

[4] Trache D., Tarchoun A.F., Derradji M., Hamidon T.S., Masruchin N., Brosse N., Hussin M.H. (2020). Nanocellulose: From fundamentals to advanced applications. Front. Chem..

[5] Sunasee R., Barchi J.J. (2021). Nanocellulose: Preparation, Functionalization and Applications. Comprehensive Glycoscience.

[6] Sunasee R., Hemraz U.D., Ckless K. (2016). Cellulose nanocrystals: A versatile nanoplatform for emerging biomedical applications. Expert Opin. Drug. Deliv..

[7] Foster E.J., Moon R.J., Agarwal U.P., Bortner M.J., Bras J., Camarero-Espinosa S., Chan K.J., Clift M.J., Cranston E.D., Eichhorn S.J. (2018). Current characterization methods for cellulose nanomaterials. Chem. Soc. Rev..

[8] Yuan Q., Bian J., Ma M.G. (2021). Advances in Biomedical Application of Nanocellulose-Based Materials: A Review. Curr. Med. Chem..

[9] Čolić M., Tomić S., Bekić M. (2020). Immunological aspects of nanocellulose. Immunol. Lett..

[10] Lam E., Hemraz U.D. (2021). Preparation and Surface Functionalization of Carboxylated Cellulose Nanocrystals. Nanomaterials.

[11] Finny A.S., Popoola O., Andreescu S. (2021). 3D-Printable Nanocellulose-Based Functional Materials: Fundamentals and Applications. Nanomaterials.

[12] Shaikh H.M., Anis A., Poulose A.M., Madhar N.A., Al-Zahrani S.M. (2022). Date-Palm-Derived Cellulose Nanocrystals as Reinforcing Agents for Poly(vinyl alcohol)/Guar-Gum-Based Phase-Separated Composite Films. Nanomaterials.

[13] Bernier A., Tobias T., Nguyen H., Kumar S., Tuga B., Imtiaz Y., Smith C.W., Sunasee R., Ckless K. (2021). Vascular and Blood Compatibility of Engineered Cationic Cellulose Nanocrystals in Cell-Based Assays. Nanomaterials.

[14] Mota R., Rodrigues A.C., Silva-Carvalho R., Costa L., Martins D., Sampaio P., Dourado F., Gama M. (2022). Tracking Bacterial Nanocellulose in Animal Tissues by Fluorescence Microscopy. Nanomaterials.

[15] Wacker M., Riedel J., Walles H., Scherner M., Awad G., Varghese S., Schürlein S., Garke B., Veluswamy P., Wippermann J. (2021). Comparative Evaluation on Impacts of Fibronectin, Heparin–Chitosan, and Albumin Coating of Bacterial Nanocellulose Small-Diameter Vascular Grafts on Endothelialization In Vitro. Nanomaterials.

[16] Imtiaz Y., Tuga B., Smith C.W., Rabideau A., Nguyen L., Liu Y., Hrapovic S., Ckless K., Sunasee R. (2020). Synthesis and Cytotoxicity Studies of Wood-Based Cationic Cellulose Nanocrystals as Potential Immunomodulators. Nanomaterials.

